# Dissection of Structure and Function of the N-Terminal Domain of Mouse DNMT1 Using Regional Frame-Shift Mutagenesis

**DOI:** 10.1371/journal.pone.0009831

**Published:** 2010-03-23

**Authors:** Leonardo D'Aiuto, Marco Marzulli, K. Naga Mohan, Ewa Borowczyk, Federica Saporiti, Andrew VanDemark, J. Richard Chaillet

**Affiliations:** 1 Department of Microbiology and Molecular Genetics, University of Pittsburgh, Pittsburgh, Pennsylvania, United States of America; 2 Department of Biological Sciences, University of Pittsburgh, Pittsburgh, Pennsylvania, United States of America; 3 Department of Neurology, University of Pittsburgh, Pittsburgh, Pennsylvania, United States of America; Temasek Life Sciences Laboratory, Singapore

## Abstract

Deletion analysis of mouse DNMT1, the primary maintenance methyltransferase in mammals, showed that most of the N-terminal regulatory domain (amino acid residues 412–1112) is required for its enzymatic activity. Although analysis of deletion mutants helps to identify regions of a protein sequence required for a particular activity, amino acid deletions can have drastic effects on protein structure and/or stability. Alternative approaches represented by rational design and directed evolution are resource demanding, and require high-throughput selection or screening systems. We developed Regional Frame-shift Mutagenesis (RFM) as a new approach to identify portions required for the methyltransferase activity of DNMT1 within the N-terminal 89–905 amino acids. In this method, a short stretch of amino acids in the wild-type protein is converted to a different amino acid sequence. The resultant mutant protein retains the same amino acid length as the wild type, thereby reducing physical constrains on normal folding of the mutant protein. Using RFM, we identified three small regions in the amino-terminal one-third of the protein that are essential for DNMT1 function. Two of these regions (amino acids 124–160 and 341–368) border a large disordered region that regulates maintenance methylation activity. This organization of DNMT1's amino terminus suggests that the borders define the position of the disordered region within the DNMT1 protein, which in turn allows for its proper function.

## Introduction

The mammalian DNA cytosine methyltransferase 1 (DNMT1) is the enzyme primarily responsible for the accurate perpetuation of DNA methylation patterns following cell division. DNMT1 is comprised of a regulatory N-terminal and a catalytic C-terminal domain, which are linked by a short stretch of Gly-Lys dipeptide repeats. The C-terminal domain (amino acid residues 1148–1620) is characterized by the presence of 10 conserved amino acid motifs, shared with many prokaryotic 5-methyl-cytosine methyltransferases [1]. The catalytic center and coenzyme binding site reside within this domain. The function of the N-terminal domain is less clear. Based primarily on prominent interacting molecules, the N-terminal domain can be divided into two separate subdomains. The more N-terminal subdomain contains the binding site for the DNA methyltransferase associated protein DMAP1 (amino acids 12–105) [2], a functional nuclear localization signal (NLS) (amino acids 191–211), and the binding site for proliferating cell nuclear antigen PCNA (amino acids 162–171) [3]. Loading of DNMT1 onto hemi-methylated DNA is mediated by SRA-domain protein UHRF [4]. The SET and RING associated (SRA) domain of UHRF recognize hemimethylated sites and directs DNMT1 to these sites [4], [5]. The fundamental role of UHRF in the maintenance of DNA methylation is demonstrated by the dramatic reduction in global CpG methylation in homozygous *Uhrf*-null ES cells and embryos. The C-terminal domain of DNMT1 contains the replication focus targeting sequence (RFTS; amino acids 350–609) [6], the zinc-binding domain, (amino acids 647–693) [7], Bromo Adjacent Homology domain 2 (BAH2; amino acids 968–1104) [8], and two additional NLS (amino acids 259–378 and 630–757) [9].

Although the essential enzymatic function of DNMT1 is the chemical conversion of a hemimethylated DNA substrate into fully methylated DNA, the regions of the protein regulating this activity have not been clearly defined. In contrast to prokaryotic methyltransferases, the C-terminal sub-domain of DNMT1 is catalytically inactive, and DNMT1 methyltransferase activity requires a substantial portion of the N-terminal domain. The direct interaction of one or more N-terminal domains with the C-terminal domain has been considered a requirement for enzymatic function [10]. The N-terminal domain may also play an important role in recognizing hemimethylated substrates. For example DNMT1-*Hha*I, a mouse prokaryotic methyltransferase hybrid containing the intact N-terminus of mouse DNMT1 and most of the coding sequence of prokaryotic *Hha*I, has a 2.5-fold preference for hemimethylated DNA over unmethylated DNA. Such preference was not observed for the parental M.*Hha*I [11]. Moreover, the parental full-length mammalian DNMT1 shows 3–11 times higher catalytic efficiency for hemimethylated DNA, suggesting that both C- and N-terminal domains are involved in distinguishing between hemimethylated and unmethylated DNA.

There have been a number of studies designed to identify the putative substrate recognition domains within the N-terminal part of DNMT1. Margot *et al*. analyzed the methyltransferase activity of a series of DNMT1 deletion mutants expressed in COS-7 cells [12]. The activity of each mutant protein was measured in a whole-cell extract as incorporation of the methyl group from S-adenosyl-L-methionine into the synthetic substrate poly(dI-dC). The 5′-most part of the analyzed N-terminal region (amino acid residues 119–425) was found to be dispensable for the methytransferase activity. In contrast, deletions within the remainder of the N-terminal domain (amino acids 426–1090) as well as in the C-terminal domain (amino acids 1091–1620) showed loss of poly(dI-dC) methylation. Although this study would suggest that the substrate recognition requires multiple motifs throughout the N-terminal and C-terminal domains of DNMT1, the large size of the analyzed deletions (ranging from amino acids 124–1088) may have precluded the identification of smaller motifs that are dispensable for activity.

The findings of subsequent studies on the role of the N-terminal region in substrate recognition conflicted with those of Margot *et al.* [12]. Araujo *et al*. [13] mapped target recognition to the same N-terminal region of the enzyme (amino acids 122–417) that was found dispensable by Margot *et al*. [12]. The Araujo *et al.* [13] findings were refuted by Fatemi *et al*. [14] who showed that this region can bind DNA but does not have the ability to distinguish between methylated and unmethylated DNA. They provided additional evidence that recognition of hemimethylated DNA is a property of the more C-terminal Zn-binding and catalytic regions. Consistent with the report by Fatemi *et al*. [14], Suetake et al. [15] reported that the DNA binding activity is located in the N-terminal amino acids 119–197. This domain does not discriminate the CG sequence and methylation status. The markedly different conclusions from these studies might be due to the different *in vitro* biochemical assays employed to measure methyltransferase activity. In none of these studies was methyltransferase activity assessed in a normal cellular context. In summary, the identification of N-terminal sub-domains responsible for target recognition and enzymatic activity remains controversial.

To more accurately address the requirement of DNMT1 regions for maintaining DNA methylation, we developed a novel mutagenesis strategy that allows a rapid and high-throughput scanning of proteins, such as DNMT1, for which structural insights into functional regions are not available. This strategy consists of site-directed mutagenesis to generate mutant cDNAs each encoding a protein that differs from the wild-type protein for the amino acid sequence of a short stretch of contiguous amino acids. The rationale of this strategy is that replacement amino acids that are tolerated at certain given positions do not play essential roles in protein structure, stability or activity. Using this approach, we show that, in contrast to previous studies of DNMT1 function, most of the mutant proteins generated by this novel approach retain methylating activity. Only frame-shifts among amino acids 124–160, 386–436, 698–740 and 792–905 abolish DNA methylation activity.

## Materials and Methods

### Generation of RFM mutants

*Dnmt1* RFM mutants were generated by site-directed mutagenesis using a plasmid in which the *Dnmt1* cDNA is transcribed from the mouse *Pgk1* promoter [16]. Site-directed mutagenesis was performed with a QuikChange site-directed mutagenesis kit (Stratagene), according to the manufacturer's instructions. The primers used for each point mutation are described in Table S1. Mutants were confirmed by DNA sequencing.

### Plasmid constructs

The pPGK-IRES-p40 plasmid was used to express some RFM mutant cDNAs from a bicistronic message; this vector has been described previously [16]. Mutants RFM4A, RFM4B, RFM10, RFM12A, RFM12B, and RFM19 cDNAs were amplified by PCR from the Pgk1 expression plasmid [16] using the primers PGKF (5′-ggg gaa ttc tac cgg gta ggg gag g-3′) and Mlu-Dnmt1R (5′-tct tcc cga cgc gtc gct agt cct tgg tag cag cct cct ctt tt-3′). PCR reactions were subjected to 25 cycles at 98°C for 30 s, 55°C for 30 s, and 72°C for 20 s, followed by a 10 min extension at 72°C using KOD DNA polymerase (Novagen). PCR products were gel-purified, digested with *Spe I* and *Mlu I*, and cloned between the *Pgk1* promoter and the IRES sequences of pPGK-IRES-p40. The integrity of the RFM mutants was verified by DNA sequencing.

### Cell cultures and transfections

Mouse embryonic stem (ES) cell lines R1 [17], *Dnmt1^c/c^* [18], and *Dnmt1^tet/tet^* [19] were used. The *C* allele of *Dnmt1* disrupts the C-terminal catalytic domain of the enzyme [18]. Transcription of the *tet* allele of *Dnmt1* is repressed by the addition of doxycycline to the culture medium [19]. ES cells were grown in DMEM supplemented with 15% fetal bovine serum, 100 µg Streptomycin/ml, 100 U Penicillin/ml, and 1000 U/ml LIF (Chemicon/Millipore). Cultures were maintained in a humidified chamber in a 5% CO_2_/air mixture at 37°C.

Transient transfections of bicistronic pPGK-IRES-p40 plasmids were carried out with Lipofectamine 2000 (Invitrogen). Cells in exponential growth were seeded (7.5×10^4^) into 24-well plates the day before transfection. Cells were transfected with 250 ng of *Dnmt1* mutant cDNAs. Forty-eight hours after transfection, supernatant was collected for measurement of IL-12 p40 concentration [16] and cells were harvested for measurements of mutant DNMT1 protein expression.

For stable expression in *Dnmt1^c/c^* ES cells, 5×10^6^ *Dnmt1^c/c^* ES cells in PBS buffer were electroporated with 20 µg each of the linearized expression vectors encoding RFM mutants along with 2 µg each of linearized pPGK-puro using the BioRad Gene Pulser II (200 V, 500 µF). Puromycin-resistant clones were picked after 8–10 days of puromycin selection (1 µg/ml), expanded using the same medium, and examined by immunoblot analysis using the anti-DNMT1 antibody UPT82 [20]. Puromycin-resistant clones of *Dnmt1^c/c^* ES cells electroporated with RFM4A-IRES-p40 and RFM12A-IRES-p40 plasmids (plus Pgk-puro) were screened for p40 expression by an ELISA assay [16]. After puromycin selection for 8–10 days, eighty clones each were grown in 48-well plates. After 4 days, the concentration of secreted IL-12 p40 protein in the medium of the transfected cells was measured using an IL-12 p40 ELISA kit (BioLegend). IL-12 p40 concentration was measured in duplicate 100-µl samples of culture medium.

For stable expression of *Dnmt1^tet/tet^* ES cells, RFM7, RFM8 and RFM9 mutant cDNAs were first cloned into a modified version of pEF1/*Myc*-His A vector (Invitrogen) in which the neomycin-resistance gene was replaced by the hygromycin-resistance gene. The expression plasmids were then linearized, electroporated into *Dnmt1^tet/tet^* ES cells, and hygromycin-resistance clones identified and expanded. For each transfected RFM mutant cDNA, twenty-four ES cell clones were cultured in the presence of 2 µg/ml doxycycline for seven days (to repress endogenous DNMT1 expression) and screened by RT-PCR and immunoblotting for expression of the mutant *Dnmt1* transcript and protein, respectively. Mutant DNMT1 protein was detected using the anti-DNMT1 UPT82 antibody [20].

### Transcription analysis

Expression of RFM7, RFM8 and RFM9 mutants in *Dnmt1^tet/tet^* ES cells was determined by RT-PCR analysis following seven days of culture of stably transfected ES cell clones with 2 µg/ml doxycycline. RNA was extracted using the RNAeasy Mini Kit (Qiagen) and followed by treatment with deoxyribonuclease to remove any residual genomic DNA. First-strand cDNA was synthesized using oligodT. The cDNA was amplified in the region between exons 6 and 17 using Dnmt1-specific oligonucleotides: Ex6F - GAG TCG GAA GAG GGG AAC TC and Ex17R - CAT GAA TTG CTT TGG CAC AC. Gel-isolated PCR products were sequenced using the Ex6F oligonucleotide.

### DNA methylation analysis

#### Southern blot analysis

Genomic DNAs from RFM mutants were digested with *Hpa*II or *Msp*I (New England Biolabs), electrophoresed on 1% agarose gels, and transferred to Genescreen nylon membrane (NEN, Boston, MA). The blots were hybridized with a ^32^P-labeled IAP probe [21]. Southern blots were washed in 2x SSC (1x SSC is 0.15 M NaCl plus 0.015 M sodium citrate) with 0.1% sodium dodecylsulfate at room temperature and with 0.1x SSC with 0.1% sodium dodecylsulfate at 65°C.

### Combined bisulfite restriction analysis (COBRA)

Genomic DNA samples were treated with sodium bisulfite using the EZ DNA methylation Gold kit (Zymo Research, USA) according to the manufacturer's recommendations. About 100 ng each of the converted DNA was amplified with primers designed for a consensus IAP LTR (GenBank accession no. M17551) [22] and the skeletal α-actin promoter (accession no. M12347). The primers used and amplification conditions were the same as previously described [23], [24]. R1 and *Dnmt1^c/c^* DNA samples were used as methylated and unmethylated controls, respectively. To assess methylation in IAP and skeletal α-actin sequences from different DNA samples, bisulfite-PCR products were digested with *Hpy*CH4IV and electrophoresed on polyacrylamide gels.

### Bisulfite genomic sequencing

IAP sequences were amplified from bisulfite treated DNA [23], cloned into TOPO TA vector (Invitrogen) and sequenced. The fraction of methylated CpGs was determined by dividing the total number of CpGs observed at eight positions of highly conserved CpGs in a total of ten sequenced IAP LTRs by 80.

### Immunoblotting

ES cells were grown in the absence of mouse embryonic fibroblast feeders and with 1,000 U of LIF/ml. Cell lysates were prepared with 10 volumes of RIPA buffer (25 mM Tris-HCl pH 7.6, 150 mM NaCl, 1% NP-40, 1% sodium deoxycholate, 0.1% SDS), denatured by heating at 95°C and then separated by electrophoresis on SDS-5% polyacrylamide gels. Afterwards, the electrophoresed proteins were transferred to PVDF membranes (Immobilon-P Millipore). DNMT1 proteins were detected using the UPT82 anti-DNMT1 antibody [20]. Membranes were blocked in 5% dry skim milk in 1X Phosphate Buffered Saline Tween-20 (PBST) for 1 hour and probed with UPT82 (1∶1,000) overnight at 4°C. Following 5 washes of 5 minutes each in PBST, the membranes were incubated for 1 hour in donkey anti-rabbit IgG (Amersham) diluted 1∶10,000 in blocking solution. Membranes were washed as above. Bound antibody was detected using the chemiluminescence detection kit ECL Plus (GE Biosciences).

### Immunocytochemistry and microscopy

Cells were fixed with 4% paraformaldehyde (PFA) for 10 min at room temperature, washed in PBS and blocked for 1 h in blocking buffer (10% goat serum in PBS). Samples were incubated with the anti-DNMT1 antibody UPT82 (1∶250 dilution) for 1 hour, washed in PBS and incubated with Texas Red-X goat anti-rabbit IgG (H+L) (Molecular Probes), and counterstained with DAPI. Images were acquired using a laser scanning confocal microscope (FluoView FV1000, Olympus).

## Results

### Generation of *Dnmt1* regional frame-shift cDNA mutants

**Figure 1:**
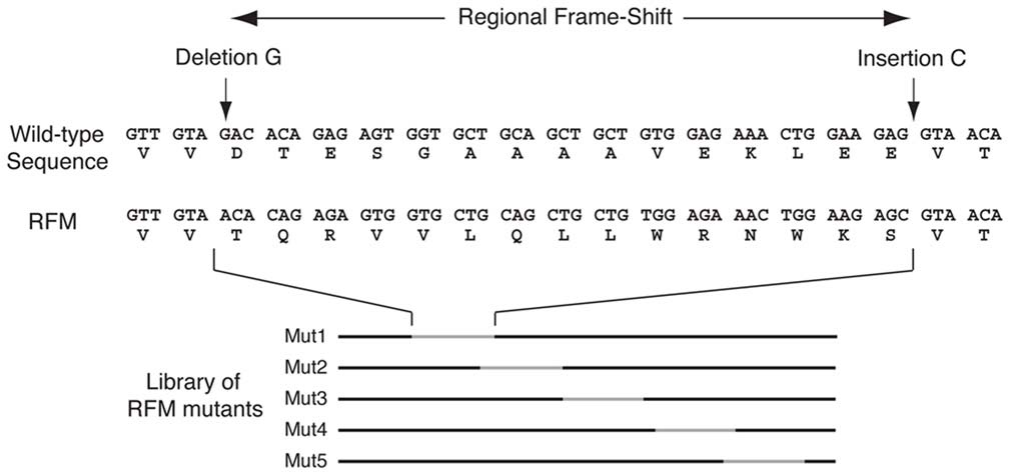
Regional Frame-Shift Mutagenesis. Through site-directed mutagenesis, a mutant cDNA carrying a nucleotide insertion at a defined site, plus a nucleotide deletion at a second defined site is generated. This results in a frame-shift from the site of the nucleotide insertion to the site of nucleotide deletion in the mutated cDNA. A library of this type of mutant is generated (Mut1–Mut5). This library encodes proteins that differ from the wild type in the amino acid sequence of a short segment.

**Figure 2:**
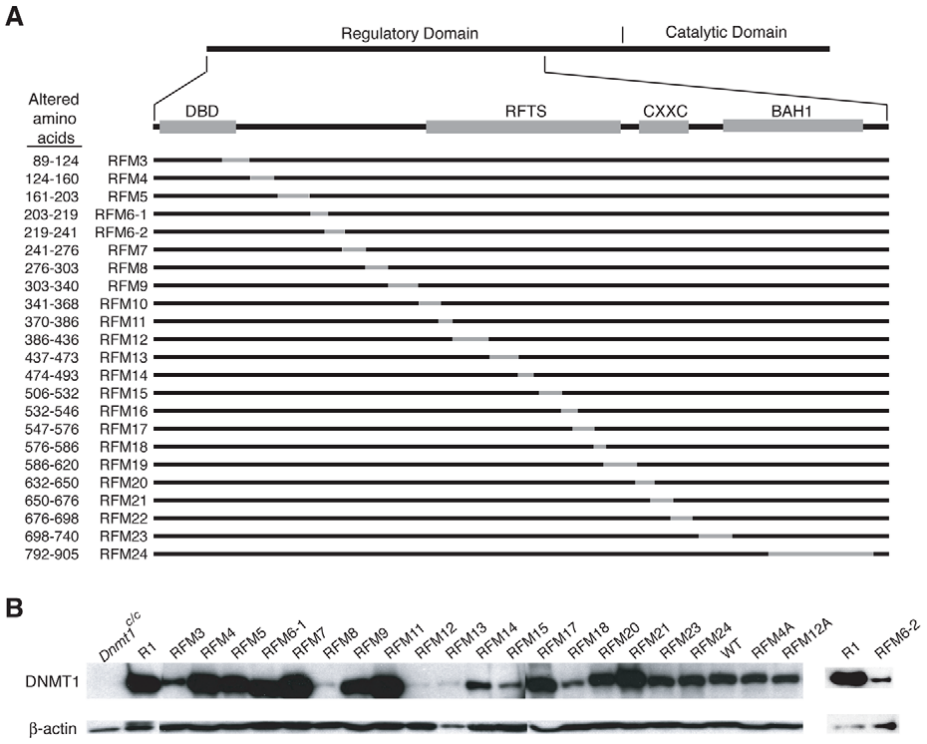
Regional Frame-Shift Mutagenesis of the DNMT1s protein. (A) Generation of DNMT1 RFM mutants. *Top*. Subdomains within the regulatory region of DNMT1. *DBD*, DMAP1 binding domain; *RFTS*, replication focus targeting sequence; *CXXC*, cysteine-rich Zn^2+^ binding motif; *BAH1*, Bromo Adjacent Homology domain 1; *Bottom*. RFM DNMT1 mutants. Stretch of amino acid sequence changed by RFM is indicated with a gray line. The RFM mutants and a wild-type *Dnmt1* cDNA (WT) were electroporated into *Dnmt1^c/c^* ES cells and restoration of DNA methylation was studied. (B) Western blotting analysis of *Dnmt1^c/c^* ES cell clones, each expressing a different RFM mutant. RFM 6-2 is shown separately.

To determine which parts of DNMT1 are required for cellular methyltransferase activity, we generated a collection of cDNAs expressing DNMT1 mutants that differ from each other in the sequence of a stretch of amino acids (Table 1). The outline of this strategy is shown in Figure 1. Using site-directed mutagenesis, a single nucleotide at a specific position in the cDNA is deleted and another nucleotide is inserted at another defined position. This results in a frame-shift of the coding sequence from the site of nucleotide insertion to the site of deletion (Figure 1). During this mutagenesis, generation of stop codons is avoided. As the resultant in-frame insertion-deletion mutations change only a portion of the protein sequence corresponding to the frame-shift, we named this strategy as Regional Frame-Shift Mutagenesis (RFM), and the mutants as RFM mutants. DNMT1 RFM mutants covering most of the N-terminal regulatory domain of DNMT1 (from the end of the DMAP1 binding domain to BAH1) were generated (Figure 2A). Different insertion and deletion sites within a region were considered and only those that result in a regional frame-shift of an average stretch of 30 amino acids were synthesized. Stability of these mutant DNMT1 proteins was tested by transient transfections into *Dnmt1^c/c^* cells devoid of detectable DNMT1 [18] and immunoblotting with the anti-DNMT1 UPT82 antibody (data not shown). All RFM mutants were expressed in *Dnmt1^c/c^* cells with the exception of RFM10 and RFM19; causes of the lack of RFM10 and RFM19 expression are addressed below.

**Table 1:** Amino acid modifications in RFM mutants.

Mutant	Amino Acid Sequence	Position
**RFM3**	**ICPWRTEHTLSLKKPTVVPPTGAGQPGEQKWQTQIA**	**89–124**
WT	DLSLENGTHTLTQKANGCPANGSRPTWRAEMADSNR	89–124
**RFM4**	**SPQDPGPSLGDPGEASRTVTPFLKLHLVPWLRGEPPG**	**124–160**
WT	RSPRSRPKPRGPRRSKSDSDTLFETSPSSVATRRTTR	124–160
**RFM4A**	**SPQDPGPSLGDPGEASRTV**	**124–142**
WT	RSPRSRPKPRGPRRSKSDS	124–142
**RFM4B**	**RTPFLKLHLVPWLRGEPPG**	**142–160**
WT	SDTLFETSPSSVATRRTTR	142–160
**RFM5**	**RPPSRLTSRRAPLNGNPRKSRKRGTRLSRLQRRETRIRNAELL**	**161–203**
WT	QTTITAHFTKGPTKRKPKEESEEGNSAESAAEERDQDKKRRVV	161–203
**RFM6-1**	**TQRVVLQLLWRNWKS**	**203–219**
WT	DTESGAAAAVEKLEE	203–219
**RFM6-2**	**QREPSWVRKSHVNRKMTTGVF**	**219–241**
WT	TAGTQLGPEEPCEQEDDNRSL	219–241
**RFM7**	**PTSHQRAIIEAEIKGGSRQRSKTGNSLGRGRGRKKG**	**241–276**
WT	RRHTRELSLRRKSKEDPDREARPETHLDEDEDGKKD	241–276
**RFM8**	**EKEVPDPGASPEIQLPNGDPRKQSQSRL**	**276–303**
WT	DKRSSRPRSQPRDPAAKRRPKEAEPEQV	276–303
**RFM9**	**ELQRLPRTETRMRGRRRDEKRHVKNWSHTPFPFRADRS**	**303–340**
WT	VAPETPEDRDEDEREEKRRKTTRKKLESHTVPVQSRSE	303–340
**RFM10**	**QKSRSKQKCDPEDQLTKVPRVWPAPRR**	**341–368**
WT	RKAAQSKSVIPKINSPKCPECGQHLDD	341–368
**RFM10A**	**QKSRSKQKCDPEDH**	**341–353**
WT	RKAAQSKSVIPKIN	341–353
**RFM10B**	**QLTKVPRVWPAPRRP**	**354–368**
WT	HSPKCPECGQHLDDP	354–368
**RFM11**	**RSTSSTLRMLWMNPRC**	**370–386**
WT	LKYQQHPEDAVDEPQM	370–386
**RFM12**	**PVRNCPSTTPPRPGLILMKILPCIGSLPSVCTAVAGTCVLSTPVSLRRKL**	**386–436**
WT	TSEKLSIYDSTSTWFDTYEDSPMHRFTSFSVYCSRGHLCPVDTGLIEKNV	386–436
**RFM12A**	**PVRNCPSTTPPRPGLILI**	**386–404**
WT	TSEKLSIYDSTSTWFDTY	386–404
**RFM12B**	**MKILPCIGSLPSVCTAVAGTCVLSTPVSLRRKL**	**404–436**
WT	YEDSPMHRFTSFSVYCSRGHLCPVDTGLIEKNV	404–436
**RFM13**	**SSTFLGVPKQFMTRIHLWKVVLMAKTSGQSISGGSVA**	**437–473**
WT	ELYFSGCAKAIHDENPSMEGGINGKNLGPINQWWLSG	437–473
**RFM14**	**LMVARRCSLASPLHLLNTF**	**474–493**
WT	FDGGEKVLIGFSTAFAEYI	474–493
**RFM15**	**RCRRKFTSARLLLSSCKTILMLYMKT**	**506–532**
WT	LMQEKIYISKIVVEFLQNNPDAVYED	506–532
**RFM16**	**RSIRLRPLFLLLPLT**	**532–546**
WT	LINKIETTVPPSTIN	532–546
**RFM17**	**CEPVHRGLPLTPRPVCSEPGRELRRSQGRY**	**547–576**
WT	VNRFTEDSLLRHAQFVVSQVESYDEAKDDD	547–576
**RFM18**	**MRPPSSCLPVC**	**576–586**
WT	DETPIFLSPCM	576–586
**RFM19**	**QSPDPFGWCLPGTEASNKARHGCYQGEGQSTHES**	**586–620**
WT	RALIHLAGVSLGQRRATRRVMGATKEKDKAPTKA	586–620
**RFM20**	**TLSSQSRLRSMIRRTRRMPL**	**632–650**
WT	DTFFSEQIEKYDKEDKENAM	632–650
**RFM21**	**RSAAAVVSVRSVSSLSVGSARRAKIWL**	**650–676**
WT	MKRRRCGVCEVCQQPECGKCKACKDMV	650–676
**RFM22**	**GSLVALDGVSRLASRGGVLTWRL**	**676–698**
WT	VKFGGTGRSKQACLKRRCPNLAV	676–698
**RFM23**	**GRRQTTMKRLMMMCQRCHHPKSCIRGRRRSRTRTASPGLGSLL**	**698–740**
WT	VKEADDDEEADDDVSEMPSPKKLHQGKKKKQNKDRISWLGQPM	698–740
**RFM24**	**RCSMRTGSALGQTQSWEPPPTPWNCSWWASAKTCSFPTSTARSRSSTKPLLKTGPWR**	**792–905**
	**EAQTLRPHCLGLRMARLTSSSSGTTRSTQGLNPHPRPSRPRTTSTSSAYLVSGWLS**	
WT	MMFHAHWFCAGTDTVLGATSDPLELFLVGECENMQLSYIHSKVKVIYKAPSENWAME	792–905
	GGTDPETTLPGAEDGKTYFFQLWYNQEYARFESPPKTQPTEDNKHKFCLSCIRLAE	

### Effect of RFM mutants on recovery of DNA methylation

To test the effect of the regional frame-shift mutations on the enzymatic activity of DNMT1, we assessed the CpG methylation levels in clones stably expressing RFM mutant proteins, and compared these levels to genomic methylation in wild-type R1 and mutant *Dnmt1^c/c^* cells. A hypomorphic *Dnmt1* allele with a small fraction (<5%) of wild-type activity maintained approximately one-third of normal genomic methylation [18], [25]. Moreover, ES cells expressing DNMT1 protein at ∼10% of normal concentration restored genomic methylation in *Dnmt1*-null ES cells [26]. For these reasons, we sought to obtain ES cells expressing mutant proteins at greater than 5% of the wild-type ES-cell DNMT1 level. *Dnmt1^c/c^* ES cells stably expressing different RFM mutants were obtained by co-electroporation of the mutant cDNA constructs with a pPGK-puromycin-resistance plasmid. After puromycin selection for 8–10 days, forty clones for each mutant construct were screened by immunoblotting. The frequency of clones expressing mutant DNMT1 proteins at levels ≥20% of the wild-type (WT) protein in R1 cells was low, ranging from three to five clones per mutant. For RFM6-2 and RFM12, only one clone each was obtained. For RFM3, RFM 8, RFM12, RFM13, RFM14, RFM15, and RFM18 only clones that express relatively low amounts of DNMT1 were obtained (Figure 2B). The reason for this is unknown, but may be due to an effect of the mutations on protein stability.

**Figure 3:**
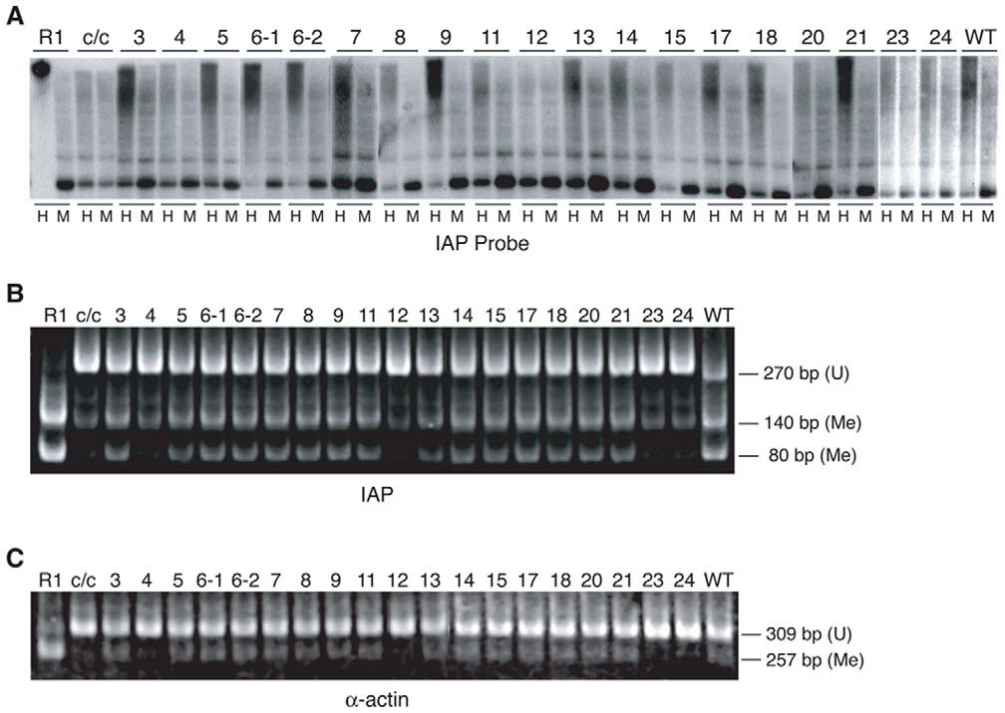
Genomic methylation assay for IAP LTR and α-actin sequences. (A) Southern blots of total DNA extracted from wild-type (R1), *Dnmt1 ^c/c^* (c/c), *Dnmt1 ^c/c^* cells expressing DNMT1 RFM mutants and *Dnmt1 ^c/c^* cells expressing wild-type DNMT1 (WT). Genomic DNA was digested with the methylation-sensitive enzyme *Hpa*II (H) and its methylation-insensitive isoschizomer, *Msp*I (M) and hybridized on a Southern blot with an IAP LTR probe. Hypomethylation of IAP LTR sequences in the *Dnmt1^c/c^* cells is indicated by hybridization to low-molecular weight DNA (1.1-kb band) in the *Hpa* II digests. (B) Methylation analysis of IAP LTR by COBRA. (C) Methylation analysis of α-actin by COBRA. PCR amplification products represent unmethylated (U) genomic DNA sequences and their digested products represent methylated (M) genomic sequences; sizes are indicated.

Genomic DNA samples from ES clones stably expressing an RFM mutant were obtained after three weeks of continuous cell culture. Samples were digested with the methylation-sensitive restriction enzymes *Hpa*II or *Msp*I, a methylation-insensitive isoschizomer, and analyzed by Southern blot hybridization using an IAP-LTR probe [21] (Figure 3). The paucity of low-molecular-weight bands following *Hpa*II digestion of the DNA from the R1 ES cells denotes a high level of DNA methylation of the IAP LTR repetitive sequences in these cells. The *Hpa*II (H) restriction pattern in the *Dnmt1^c/^* ^c^ cells was distinct from that of R1 cells by an increased hybridization of low-molecular-weight bands, indicating a strong reduction in DNA methylation. The *Hpa*II digestion pattern of *Dnmt1^c/c^* cells differs slightly from the *Msp*I digestion pattern, indicating a low level of DNA methylation at the IAP LTR sequences, probably due to the activity of the *de novo* methyltransferases [27]. Introduction of most *Dnmt1* RFM cDNA mutants into *Dnmt1^c/c^* cells resulted in a partial restoration of methylation of bulk repetitive DNA to varying degrees. This was observed as a substantial increase in the hybridization to high-molecular-weight DNA, accompanied by a reduction in the intensity of the low-molecular-weight bands in the *Hpa*II digests (Figure 3A). These results indicate that *Dnmt1^c/c^* ES cells expressing RFM4, RFM12, RFM23, or RFM24 did not recover methylation of their IAP LTRs. RFM4, RFM23 and RFM24 cells expressed approximately wild-type levels of DNMT1 protein (Figure 2B), indicating that their restoration defect was not likely due to inadequate protein. For RFM 12 however, we could not exclude the possibility that its inactivity was due to the low level of mutant protein expression (Figure 2B).

We also used combined bisulfite restriction analysis (COBRA) to measure the level of IAP LTR and α-actin promoter methylation among the DNA samples to identify mutants that fail to maintain methylation. The single-copy skeletal α-actin promoter is normally partially methylated both *in vivo* in day-8.5 mouse embryos [24] and in mouse ES cells [28]. In these assays, genomic DNA was treated with sodium bisulfite to convert unmethylated cytosines to uracils. Methylated cytosines are resistant to such conversion. As a result, when bisulfite–converted DNA is amplified with primers specific to a highly conserved IAP LTR sequence and to the α−actin promoter, methylated sequences retain CpGs whereas unmethylated sequences do not contain CpGs. On this basis, digestion of bisulfite PCR products by *Hpy*CH4IV (recognition site ACGT) indicates methylation and resistance to digestion indicates absence of methylation. As shown in Figures 3B and 3C, there is a greater extent of digestion of the bisulfite-PCR products for both IAP-LTR and α−actin promoter in R1 cells than in the *Dnmt1^c/c^* cells, consistent with these sequences being methylated in R1 cells and significantly unmethylated in the *Dnmt1^c/c^* cells. When *Dnmt1^c/c^* clones expressing wild type DNMT1 were studied, there was a clear difference in the extent of digestion of the two categories of sequences from the *Dnmt1^c/c^* cells, indicating a restoration of methylation in these cells by the wild-type enzyme. A similar restoration was observed for clones expressing most of the RFM mutants. Only RFM4, RFM12, RFM23 and RFM24 did not restore genomic methylation. The COBRA assay results (Figure 3B) agree with the results of the Southern blot hybridizations (Figure 3A).

To obtain a more quantitative assessment of the level of restored DNA methylation in cells expressing RFM mutants, we performed bisulfite genomic sequencing on a subset of RFM mutants to determine their level of IAP methylation (Table 2). In these experiments ten IAP alleles for each mutant were analyzed for the presence of methylated CpGs. In agreement with Southern and COBRA analysis we observed a significant increase in the number of methylated CpGs among sequences obtained from clones containing RFM mutants 9, 11, 15 and 21. This extent of increase is similar to that observed in *Dnmt1^c/c^* cells expressing wild-type DNMT1. Significant differences among RFM mutants 4, 12, 23, 24 and *Dnmt1^c/c^* cells were not observed.

**Table 2:** Determination of IAP methylation by bisulfite genomic sequencing.

Cell line	% methylated CpG dinucleotides^a^^,^ b
*Dnmt1^c/c^*	10.0
*Dnmt1^c/c^* + WT DNMT1s	47.5
*Dnmt1^c/c^* + RFM4	8.3
*Dnmt1^c/c^* + RFM9	38.9
*Dnmt1^c/c^* + RFM11	32.4
*Dnmt1^c/c^* + RFM12	10.1
*Dnmt1^c/c^* + RFM15	33.3
*Dnmt1^c/c^* + RFM21	42.5
*Dnmt1^c/c^* + RFM23	10.1
*Dnmt1^c/c^* + RFM24	11.0

a The percent of methylated CpG dinucleotides for each cell line was determined by sequencing ten IAP alleles amplified from bisulfite-treated genomic DNA. Because of their somewhat divergent nature, the IAP LTR sequences contain 7 to 12 CpGs and most of them contain eight CpGs. Therfore, methylation was assessed at the eight highly conserved CpG dinucleotide positions; CpGs at these positions in the sequence of the bisulfite-converted (sense) strand was scored as methylated CpG and TpGs scored as unmethylated CpGs.

b The efficiency of bisulfite conversion was 100%, based on the absence of CpA, CpC and CpT dinucleotides in the sequence of the bisulfite-converted (sense) strand.

**Figure 4:**
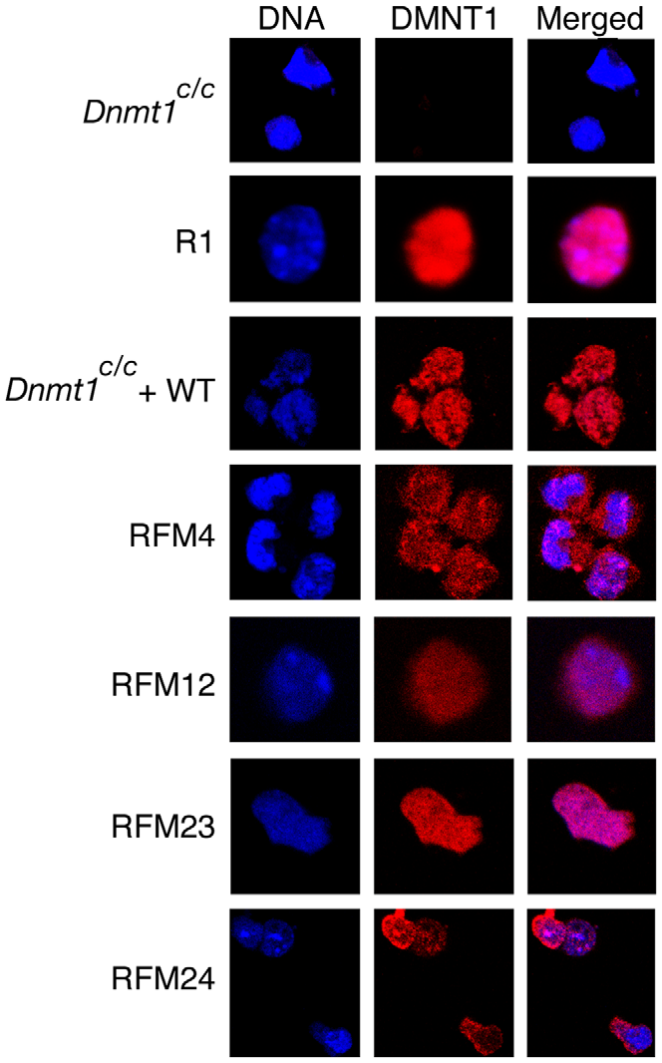
Immunolocalization of wild-type (WT) and RFM mutants expressed in *Dnmt1^c/c^* ES cells. R1 and *Dnmt1^c/c^* cells are shown for comparison. DNA column shows DAPI-stained cells. DNMT1 column shows UPT82-stained cells.

To further characterize RFM mutants that do not restore DNA methylation, cellular localizations of mutant DNMT1 proteins in *Dnmt1^c/c^* ES cells expressing RFM4, RFM12, RFM23 and RFM24 were determined. Confocal images showed that DNMT1 in RFM4 is partially retained in the cytoplasm (Figure 4). RFM12 showed a distribution similar to RFM4. DNMT1 in RFM23 and RFM24 showed only nuclear localization (Figure 4). A concentration gradient of DNMT1 was observed in the nuclei of RFM24, with a higher concentration of the protein at the nuclear periphery. We conclude from these observations that the lack of function of RFM4, RFM12, RFM23 and RFM24 is not due to the inability to accumulate in the nucleus of ES cells.

### Further analysis of faulty RFM mutants

**Figure 5:**
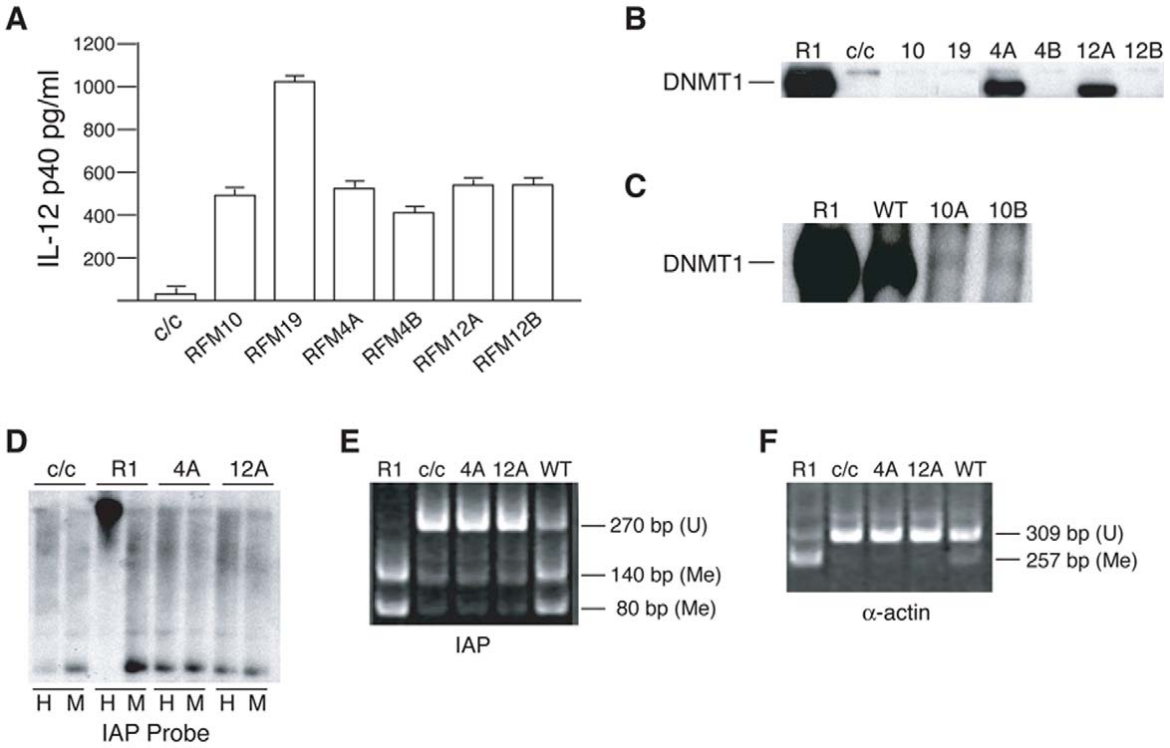
Further characterization of RFM mutants RFM10, RFM19, RFM4 and RFM12. (A) Level of IL-12 p40 protein expression from *Dnmt1^c/c^* ES cells transiently expressing a bicistronic mRNA from the pPGK-IRES-p40 plasmid. cDNAs encoding RFM mutants RFM10, RFM19, RFM4A, RFM4B, RFM12A and RFM12B were cloned between the PGK promoter and the IRES sequence. RFM4A and RFM4B are sub-frame-shifts of RFM4 and RFM12A and RFM12B are sub-frame-shift mutants of RFM12. (B) Immunoblot depicting mutant protein expression in transiently transfected *Dnmt1^c/c^* ES cells compared to wild-type R1 and untransfected *Dnmt1^c/c^* (c/c) ES cells. (C) Immunoblot depicting mutant protein expression in transiently transfected *Dnmt1^c/c^* ES cells compared to R1 ES cells and to *Dnmt1^c/c^* ES cells transiently expressing wild-type DNMT1 protein (WT). (D) Southern blot of stably expressing RFM4A and RFM12A clones hybridized to an IAP LTR probe. The levels of mutant protein expression in these clones is shown in Figure 2B. (E) Methylation analysis of IAP LTR sequences in stably expressing ES cells by COBRA. (F) Methylation analysis of the α-actin promoter in stably expressing ES cells by COBRA.

Lack of detectable DNMT1 protein in *Dnmt1^c/c^* ES cells containing RFM10 and RFM19 may be due to degradation of the mutant *Dnmt1* mRNAs or proteins. To explore this possibility, RFM10 and RFM19 mutant cDNAs were cloned into pPGK-IRES-p40, an IRES-based bicistronic vector that uses the human interleukin 12 (IL-12) p40 cDNA as a reporter gene [16]. The resulting constructs were named pPGK-RFM10-IRES-p40, and pPGK-RFM19-IRES-p40. The first cistron, encoding the DNMT1 mutant is translated by a cap-dependent mechanism, whereas the second cistron encoding IL-12 p40 requires translation by the IRES. These constructs were transiently transfected into *Dnmt1^c/^* ^c^ ES cells. IL-12 p40 and DNMT1 expression were assayed 48 hours after transfection by ELISA and immunoblotting, respectively. IL-12 p40 was expressed from both bicistronic mRNAs (Figure 5A). However, no RFM10 and RFM19 proteins were seen (Figure 5B), presumably due to degradation of these mutant proteins in ES cells.

RFM mutant 4 (RFM4) and RFM mutant 12 (RFM12) were expressed in *Dnmt1^c/c^* cells, but did not restore genomic methylation (Figures 2 and 3). To analyze these mutants further, additional rounds of RFM were performed to divide each mutant into adjacent and non-overlapping smaller RFM mutants, thus obtaining sub-mutants 4A and 4B (for RFM4), and sub-mutants 12A and 12B (for RFM12) (see Table 1 for the amino-acid sequences of the sub-mutants). The stability of these new sub-mutant proteins was studied using the pPGK-IRES-p40 vector. pPGK-RFM4A-IRES-p40, pPGK-RFM4B-IRES-p40, pPGK-RFM12A-IRES-p40, and pPGK-RFM12B-IRES-p40 were transiently transfected in *Dnmt1^c/c^* ES cells. After 48 hours, IL-12 p40 was expressed from all bicistronic mRNAs (Figure 5A), whilst DNMT1 protein expression was observed with RFM4A and RFM12A, but not with RFM4B and RFM12B (Figure 5B). These results indicate the RFM4B and RFM12B sub-mutants undergo degradation. The observation that only poorly expressing RFM12 clones were obtained (Figure 2B) may be related to this presumed RFM12B degradation. *Dnmt1^c/c^* ES cell clones stably expressing RFM4A and RFM12A were then established, and the level of CpG methylation of IAP LTR and α-actin promoter sequences was assessed after three weeks of continuous culture of these clones. As shown in Figures 5D–5F, these sub-mutant proteins were unable to maintain genomic methylation.

RFM mutants immediately N- or C-terminal of unstable RFM10 are expressed as stable proteins that restore methylation in *Dnmt1^c/c^* ES cells. To determine if a portion of RFM10 would also result in a stable RFM mutant protein, two additional rounds of RFM were performed to generate sub-mutants RFM10A and RFM10B. When these cDNAs were transiently expressed from the *Pgk-1* promoter in *Dnmt1^c/c^* ES cells, no mutant protein expression was observed, although transient expression of a control wild-type *Dnmt1* cDNA resulted in robust expression of the wild-type DNMT1 protein (Figure 5C). These findings indicate that most or all of DNMT1 defined by RFM10 requires a specific amino acid sequence for overall DNMT1 stability and function.

### Maintenance methylation activity in RFM mutants

**Figure 6:**
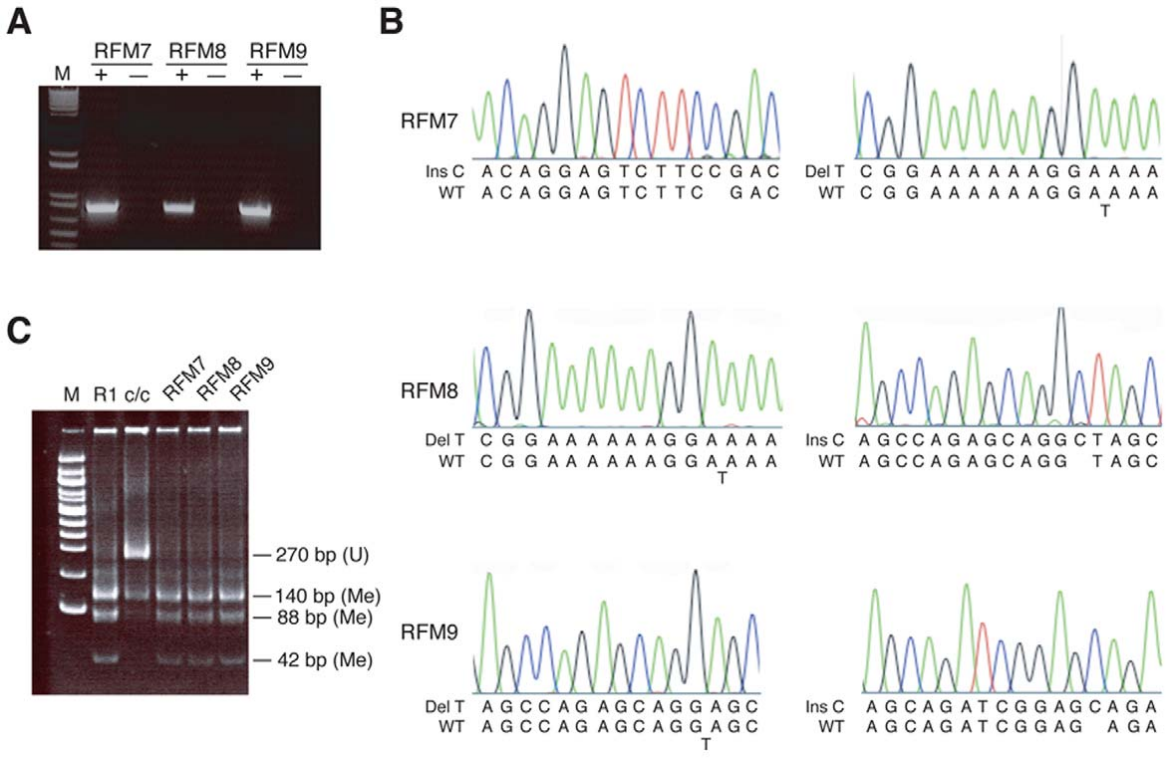
RFM7, RFM8 and RFM9 mutant proteins maintain genomic methylation in *Dnmt1^tet/tet^* ES cells. (A) RT-PCR analysis of exogenous RFM7, RFM8 and RFM9 mRNA expression in *Dnmt1^tet/tet^* ES cells treated with doxycycline to extinguish endogenous *Dnmt1* expression. + indicates presence of reverse transcriptase (RT) in cDNA synthesis reaction; - indicates absence of RT in cDNA synthesis reaction. (B) Sequence identification of nucleotide deletions (del) and insertions (ins) in RT-PCR products shown in panel A. The corresponding wild-type (WT) sequences were obtained from RT-PCR products of endogenous *Dnmt1* mRNA present in *Dnmt1^tet/tet^* ES cells cultured in the absence of doxycycline. (C) COBRA of IAP LTR sequences in *Dnmt1^tet/tet^* ES cells expressing RFM7, RFM8 and RFM9.

The majority of analyzed RFM mutants restored genomic methylation in *Dnmt1^c/c^* ES cells. Three of these mutants, RFM7, RFM8 and RFM9 were also evaluated for their ability to maintain already established genomic methylation in *Dnmt1^tet/tet^* ES cells [19]. *Dnmt1^tet/tet^* ES cells have genetically engineered TET-OFF *Dnmt1* alleles that are transcriptionally silenced in the presence of doxycycline. Following transfection with a plasmid expressing the hygromycin-resistance gene and an RFM mutant, hygromycin-resistant ES clones were screened for expression of the RFM mutant protein after seven days of exposure to 2 µg doxycycline, which extinguished endogenous DNMT1 expression. RNA from each doxycycline-treated clone expressing a DNMT1 mutant (data not shown) was subjected to RT-PCR analysis (Figure 6A), which confirmed that only the mutant *Dnmt1* was transcribed (Figure 6B). IAP sequences were methylated at wild-type levels in *Dnmt1^tet/tet^* cells that express RFM7, RFM8 or RFM9 (Figure 6C). Thus, as *Dnmt1^tet/tet^* ES cells switch from expressing wild-type DNMT1 to expressing just RFM7, RFM8 or RFM9, genomic methylation is maintained. We conclude from this analysis that restoration of genomic methylation by an RFM mutant in *Dnmt1^c/c^* ES cells corresponds to the protein's ability to maintain genomic methylation in the absence of wild-type DNMT1.

## Discussion

### RFM analysis of protein structure and function

An accurate and complete dissection of protein structure and function would require an analysis of the structural and functional roles of amino acid residues in the protein of interest. This goal is achieved by comparing the wild-type protein with a mutant protein carrying amino acid changes. To obtain specific mutant proteins, a number of different approaches have been engaged. These fall into the two main categories of rational and random methods. Rational methods can be applied to a relatively small class of proteins for which a model of structure-function relationship has been established [29]. However, these methods often meet with limited success due to our inability to completely infer function from structure [30]. Further, these methods cannot be applied to proteins such as DNMT1 where the relationship between structure and function is largely unknown. Random mutagenesis allows the characterization of proteins for which a structure–function relationship model does not exist. One of the limitations of random mutagenesis is the necessity to generate a large collection of protein variants, which in turn requires efficient and rapid protocols for screening and/or selection of variants with the desired phenotypes. Therefore, any standard random mutational approach to analyze DNMT1 function would be extremely laborious. Deletion analysis of multidomain proteins has been effectively used to rapidly identify large domains within a protein that are dispensable for a particular activity. However, certain deletions may severely affect protein structure or stability, and therefore preclude the identification of functionally important amino acid residues [31]. Amino acid substitutions on the other hand are likely to be better tolerated than deletions in the same region [22]. These reports suggest that additional methods are required to rapidly investigate the relationship among protein sequence, structure and function.

We developed a new mutagenesis strategy in which the sequence of a short stretch of amino acids in the wild-type protein is changed by nucleotide insertion and deletion at defined sites. This results in a frame-shift from the site of nucleotide insertion to the site of nucleotide deletion. The normal reading frame is maintained outside these nucleotide changes. Although a mutant protein obtained by RFM carries several amino acid changes, it retains the same overall length as the wild-type protein. Because this method was primarily designed to produce and analyze a series of frame-shift mutants along the protein's length, we termed this strategy Regional Frame-Shift Mutagenesis (RFM). We anticipate that the majority of such frame-shifts will be better tolerated than deletions in the same regions and that only a minority of frame-shifts will disrupt protein function. We demonstrated the feasibility of this method to identify *in situ* portions of DNMT1 required for maintenance methylation activity within N-terminal amino acids 89–905.

### RFM reveals important features of DNMT1 function

**Figure 7:**
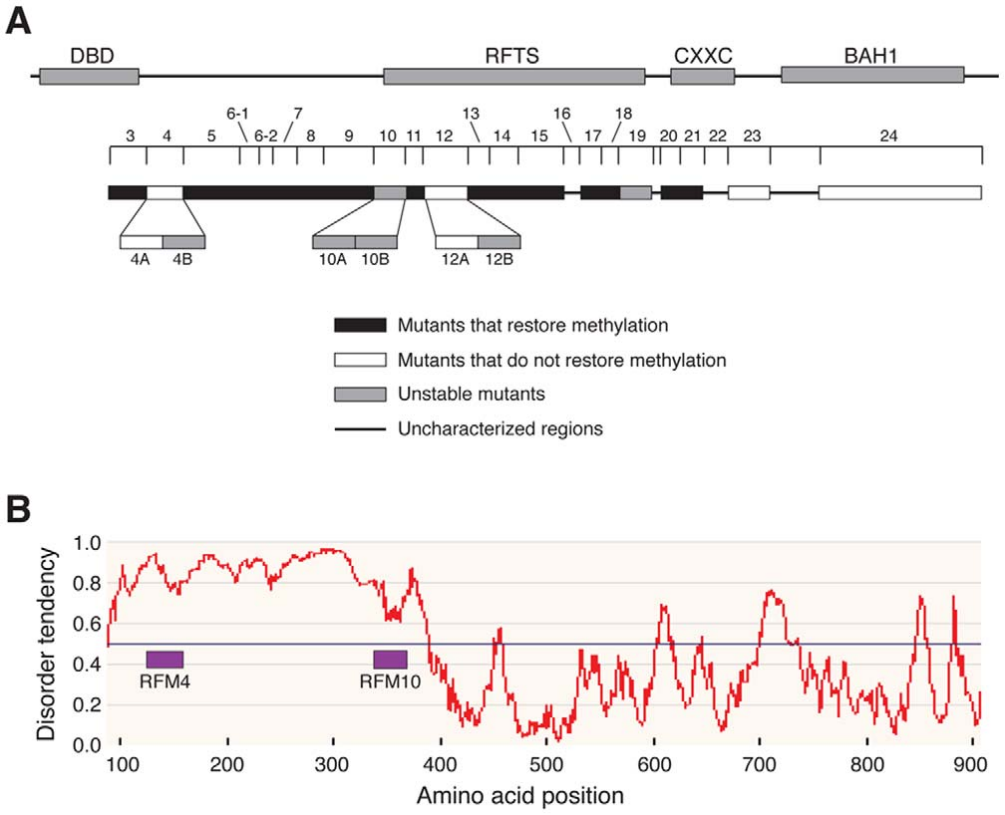
Organization of the amino terminus of DNMT1. A. Summary of the effects of regional frame-shift mutagenesis on different regions of the N-terminal 880 amino acids of DNMT1. Numerals indicate the different RFM mutants. B. Disordered protein prediction score for the amino terminal portion of DNMT1. The plot was generated using the IUPred disorder prediction algorithm (iupred.enzim.hu); the calculated degree of disorder from amino acids 89–905 is plotted. Amino acid positions are aligned with the diagram in panel A, and the positions of RFM4 and RFM10 within the disordered region are shown.

Fourteen out of 19 RFM mutants generated within amino acids 89–905 restored methylation in *Dnmt1^c/c^* cells, suggesting that most of the N-terminal region is tolerant of amino acid substitutions. Four RFM mutants (RFM 4, RFM12, RFM23, and RFM24) did not restore methylation (Figure 7A). Of these, RFM4 and RFM12 contain frame-shifts from amino acids 124–160 and 386–436 respectively. This result is not in agreement with Margot *et al*. who reported that a truncated version of DNMT1 lacking amino acids 119–425 retained catalytic activity [12]. Therefore, RFM has resulted in the identification of a short stretch that is functionally important in a region of DNMT1 that was considered as dispensable. Previous studies have shown that the RFTS domain (amino acids 350–609) is indispensable for DNMT1 enzymatic activity. We generated 11 RFM mutants in this region (RFM9-RFM19). Of these, only RFM12 (amino acids 386–436) lacks enzymatic activity. RFM10 (amino acids 341–368) and RFM19 (amino acids 586–620), are transcribed but not expressed, indicating that these proteins may be abnormally folded and degraded. Since the remaining RFM mutants are capable of restoring genomic methylation, our results suggest that the central portion of the RFTS domain (amino acids 437–586) is tolerant to amino acid substitutions. In agreement with previous reports, RFM23 and RFM24 encompassing BAH1 are defective in maintenance of methylation. In summary, our approach identified new regions in DNMT1 that are functionally important, and also ruled out regions that were previously suggested to be indispensable.

RFM4, RFM10 and RFM12 were studied further with additional rounds of RFM. RFM4B and RFM12B were transcribed but not expressed in *Dnmt1^c/c^* ES cells, indicating that these mutants may fail to assume their proper conformation and therefore be degraded by intracellular proteolytic systems. In contrast, RFM4A and RFM12A were expressed but defective in maintenance methylation. RFM10 is also transcribed but not expressed, and additional rounds of RFM showed that the sub-mutants RFM10A and RFM10B were also not expressed. These results indicate that in some instances, exemplified by RFM4 and RFM12, further RFM analysis can lead to refinements that better define both functionally and structurally important regions of the parent wild-type DNMT1 protein. Mutations in RFM4A (amino acids 124–142) and RFM12A (amino acids 386–404) do not affect the stability of the mutant protein, but inactivate protein function (functionally important), whereas mutations in RFM4B (amino acids 142–160) and RFM12B (amino acids 405–436) result in degradation of these proteins presumably due to misfolding (structurally important).

Notably, some of the clones studied expressed very low amounts of mutant DNMT1 proteins (Figure 2B). RFM13 was observed to restore methylation in the *Dnmt1^c/c^* cells despite the low level of expression, suggesting that there is no strict relationship between the levels of expression of DNMT1 with the levels of restoration of DNA methylation.

The inactive RFM4 and RFM10 mutants are located in the N- and C-terminal boundaries of a large predicted disordered region extending from amino acid ∼100 to amino acid ∼400 (Figure 7B). Within this disordered region there is a mammal-specific region that regulates the maintenance of methylation on different DNA sequences [19]. Most disordered regions interact with protein or DNA, and may acquire ordered structure upon binding to specific proteins, DNA sequences or ligands. The conformational flexibility of disordered regions allows them to interact efficiently with several different target molecules [32]. In this regard, the predicted disordered amino-terminal region of DNMT1 binds several proteins, including Rb and MeCP2 [33] and most likely interacts with DNA [13]. We speculate that the regions defined by RFM4 and RFM10 help to define the functionally important disordered region, possibly by fixing the location of the disordered region relative to other regions of DNMT1. In addition, RFM10, RFM12 and RFM19 may identify regions of DNMT1 that are important for DNMT1 dimerization [34].

Disagreements between some of our results and previously published data might be explained by the difference in mutagenesis strategies used to dissect the DNMT1 N-terminal domain. First, the size of deletions analyzed by Margot *et al*. [12] were large, and because of this small regions of DNMT1 that are dispensable for function were likely not identified. Second, in the same study, maintenance methyltransferase activity was measured as the extent of incorporation of methyl groups from S-adenosyl methionine (SAM) into poly(dI-dC), a synthetic substrate that interacts with Dnmt1 in a non-physiological manner [23]. Other studies also used synthetic substrates. For example, Araujo *et al*. [13] studied binding of different domains of DNMT1 using solid-state hemimethylated DNA substrates, and in another study DNA binding of various domains of DNMT1 was assessed using unmethylated and hemimethylated oligonucleotides [14]. All of these studies evaluated DNMT1 action *in vitro* rather than on chromosomal DNA in a cellular environment. Lastly, regions important for the DNMT1 stability cannot be identified in an *in vitro* study. Thus, the application of regional frame-shift mutagenesis to the study of intracellular function of the DNMT1 protein yielded some important findings that were not revealed by the more commonly used genetic and biochemical approaches.

In summary, RFM is a novel and efficient mutagenesis strategy that enables rapid generation of a large number of mutant proteins that differ from the wild-type protein in the amino acid sequence of a short segment. This method is likely to preserve structural and functional integrity of protein outside the mutated region and also appears to be an attractive approach to the study of large proteins (such as DNMT1) in which a model of structure-function relationship has not been established. RFM mutagenesis will provide a useful complementary approach for scanning proteins to quickly identify those regions carrying fundamentally important information for protein folding, stability or activity.

## Supporting Information

Table S1 Primers used to generate RFM mutants
(0.06 MB DOC)
