# Supplementary material for: Dissection of Structure and Function of the N-Terminal Domain of Mouse DNMT1 Using Regional Frame-Shift Mutagenesis
Source: PLoS One. 2010 Mar 23;5(3):e9831. doi: 10.1371/journal.pone.0009831 (PMC2843745; doi:10.1371/journal.pone.0009831)
Supplement: Table S1 — Primers used to generate RFM mutants (0.06 MB DOC) [file pone.0009831.s001.doc]

Table S1. Primers used to generate RFM mutants

| **RFM3 del** | 5’agtccctcttaaataag_atttgtccttggagaac 3’ | **RFM3 ins** | 5’atggcagactcaaatag**c**atccccaagatccaggcc 3’ |
| --- | --- | --- | --- |
| **RFM4 del** | 5’atggcagactcaaatag_tccccaagatccaggcc 3’ | **RFM4 ins** | 5’acgaggagaaccaccagg**c**cagaccaccatcacggc 3’ |
| **RFM4A del** | 5’atggcagactcaaatag_tccccaagatccaggcc 3’ | **RFM4A ins** | 5’gaagcaagtcggacagt**c**gacaccctttcagttga 3’ |
| **RFM4B del** | 5’gaagcaagtcggacag_gacaccctttcagttga 3’ | **RFM4B ins** | 5’acgaggagaaccaccagg**c**cagaccaccatcacggc 3’ |
| **RFM5 del** | 5’acgaggagaaccaccagg_agaccaccatcacggc 3’ | **RFM5 ins** | 5’taagaaacgcagagttg**c**tagacacagagagtggt 3’ |
| **RFM6-1 del** | 5’aagaaacgcagagttgta_acacagagagtggt 3’ | **RFM6-1 ins** | 5’tggagaaactggaagag**c**gtaacagcgggaacccag 3’ |
| **RFM6-2 del** | 5’gagaaactggaagaggta_cagcgggaacccagct 3’ | **RFM6-2 ins** | 5’atgacaacaggagtctt**c**cgacgtcacaccagaga 3’ |
| **RFM7 ins** | 5’atgacaacaggagtctt**c**cgacgtcacaccagaga 3’ | **RFM7 del** | 5’gaggacggaaaaaagga_aaaagaagttccagacc 3’ |
| **RFM8 del** | 5’gaggacggaaaaaagga_aaaagaagttccagacc 3’ | **RFM8 ins** | 5’gtcagagccagagcagg**c**tagctccagagactccga 3’ |
| **RFM9 del** | 5’gtcagagccagagcagg_agctccagagactccga 3’ | **RFM9 ins** | 5’ttcagagcagatcggag**c**agaaaagccgctcaaag 3’ |
| **RFM10 ins** | 5’ttcagagcagatcggag**c**agaaaagccgctcaaag 3’ | **RFM10 del** | 5’cagcacctagacgaccc_aacctgaagtaccagca 3’ |
| **RFM10A ins** | 5’ttcagagcagatcggag**c**agaaaagccgctcaaag 3’ | **RFM10A del** | 5’agtgtgatcccgaagatc_actcaccaaagtgcccc 3’ |
| **RFM10B ins** | 5’agtgtgatcccgaagatc**C**aactcaccaaagtgcccc 3’ | **RFM10B del** | 5’cagcacctagacgaccc_aacctgaagtaccagca 3’ |
| **RFM11 del** | 5’cctagacgaccctaacc_gaagtaccagcagcacc 3’ | **RFM11 ins** | 5’ggatgaaccccagatgt**c**tgaccagtgagaaactg 3’ |
| **RFM12 del** | 5’ggatgaaccccagatgtt_accagtgagaaactgtc 3’ | **RFM12 ins** | 5’gtctcattgagaagaatg**c**tagagctctacttttct 3’ |
| **RFM12A del** | 5’ggatgaaccccagatgtt_accagtgagaaactgtc 3’ | **RFM12A ins** | 5’cctggtttgatacttat**c**gaagattctcccatgca 3’ |
| **RFM12B del** | 5’tcgacctggtttgatact_atgaagattctcccatgcat 3’ | **RFM12B ins** | 5’gtctcattgagaagaatg**c**tagagctctacttttct 3’ |
| **RFM13 del** | 5’tcattgagaagaatgta_agctctacttttctgg 3’ | **RFM13 ins** | 5’agtggtggctcagtggc**c**tttgatggtggcgagaa 3’ |
| **RFM14 del** | 5’tggtggctcagtggctt_gatggtggcgagaa 3’ | **RFM14 ins** | 5’atttgctgaatacattt**c**tgatggagcccagcaaa 3’ |
| **RFM15 del** | 5’tgagccaatatttgggc_gatgcaggagaaaattt 3’ | **RFM15 ins** | 5’tgctgtatatgaagacc**c**tgatcaataagattgag 3’ |
| **RFM16 del** | 5’tgctgtatatgaagacc_gatcaataagattgagaccac 3’ | **RFM16 ins** | 5’ctccttctaccattaa**c**tgtgaaccggttcacag 3’ |
| **RFM17 ins** | 5’ctccttctaccattaa**c**tgtgaaccggttcacag 3’ | **RFM17 del** | 5’gaagccaaggacgat_atgagacccccatc 3’ |
| **RFM18 del** | 5’gaagccaaggacgat_atgagacccccatc 3’ | **RFM18 ins** | 5’tctccctgtatg**c**agagccctgatccatttg 3’ |
| **RFM19 ins** | 5’tctccctgtatg**c**agagccctgatccatttg 3’ | **RFM19 del** | 5’cacccacgaaag_caccaccaccaagc 3’ |
| **RFM20 del** | 5’gtctatcagatcttt_acactttcttctca 3’ | **RFM20 ins** | 5’aaggagaatgcca**c**tgaagcgccgccgctgt 3’ |
| **RFM21 del** | 5’aaggagaatgcca_gaagcgccgccgctgt 3’ | **RFM21 ins** | 5’gcgtgcaaagatatgg**c**tgaagtttggtggcact 3’ |
| **RFM22 del** | 5’ggcgtgcaaagatatgg_gaagtttggtggcact 3’ | **RFM22 ins** | 5’gtgtcctaacttggcgg**c**tgaaggaggcagacgac 3’ |
| **RFM23 del** | 5’tgtcctaacttggcgg_gaaggaggcagacgac 3’ | **RFM23 ins** | 5’tggcttgggcagccta**c**tgaagattgaagagaat 3’ |
| **RFM24 del** | 5’agacaaaaatggtcaga_gatgttccatgcgcactg 3’ | **RFM24 ins** | 5’tatccggctggctgagc**c**tgagacaaaaagaaatgc 3’ |
